# What determines adult cognitive skills? Influences of pre-school, school, and post-school experiences in Guatemala

**DOI:** 10.1007/s40503-014-0004-4

**Published:** 2014-02-13

**Authors:** Jere R. Behrman, John Hoddinott, John A. Maluccio, Erica Soler-Hampejsek, Emily L. Behrman, Reynaldo Martorell, Manuel Ramírez-Zea, Aryeh D. Stein

**Affiliations:** Department of Economics, Sociology and Demography, University of Pennsylvania, Philadelphia, PA, USA; Department of Poverty, Health, and Nutrition Division, International Food Policy Research Institute, Washington, DC, USA; Department of Economics, Middlebury College, Middlebury, VT, USA; Population Council, New York, NY, USA; Biology, University of Pennsylvania, Philadelphia, PA, USA; Global Health, Emory University, Atlanta, GA, USA; Institute of Nutrition for Central America and Panama (INCAP), Guatemala City, Guatemala; Global Health, Emory University, Atlanta, GA, USA

**Keywords:** Cognitive skills, Schooling, Guatemala, Nutrition

## Abstract

Most empirical investigations of the effects of cognitive skills assume that they are produced by schooling. Drawing on longitudinal data to estimate production functions for adult verbal and nonverbal cognitive skills, we find that: (1) School attainment has a significant and substantial effect on adult verbal cognitive skills but not on adult nonverbal cognitive skills; and (2) Pre-school and post-school experiences also have substantial positive significant effects on adult cognitive skills. Pre-school experiences captured by height for age at 6 years substantially and significantly increase adult nonverbal cognitive skills, even after controlling for school attainment. Post-school tenure in skilled jobs has significant positive effects on both types of cognitive skills. The findings (1) reinforce the importance of early life investments; (2) support the importance of childhood nutrition (“Flynn effect”) and work complexity in explaining increases in nonverbal cognitive skills; (3) call into question interpretations of studies reporting productivity impacts of cognitive skills that do not control for endogeneity; and (4) point to limitations in using adult school attainment alone to represent human capital.

## 1 Introduction

Increasing the stock of human capital is central to expanding individual options and to economic development. One dimension of human capital, cognitive skills—the capacity to assess and solve problems, or in Schultz’s (1975) memorable phrase, “the ability to deal with disequilibria” is widely believed to affect productivity in many activities, including work, raising children, and improving one’s own and others’ health and nutrition. Substantial empirical literature, mostly based on the relationship between school attainment and the outcomes of adult activities, is interpreted in support of these possible influences.^1^

Consequently, it is critical to understand the processes by which dimensions of adult human capital, such as cognitive skills, are determined. This may seem to be well-trodden territory, as there are hundreds of studies of the determinants of schooling.^2^ But school attainment is not necessarily a direct measure of adult cognitive skills. Instead, it can be considered one of the inputs, though likely an important one, into the production of those skills. Moreover, in developing countries, schooling typically is limited to particular periods of individuals’ lives, childhood and adolescence. Other experiences, both before and after one’s school years, also may affect cognitive skills. For example, much literature emphasizes the importance of nutrition from conception onward for neural and cognitive development.^3^ There is also a substantial literature that emphasizes the importance of post-school experiences, particularly in the labor market, in determining adult cognitive skills or in determining productivity and wages, which usually are interpreted as reflecting such skills.^4^ If pre-school or post-school experiences have significant impacts on the cognitive skills of adults and are correlated with schooling—as is likely if there is correlation among human capital investments across life-cycle stages—analyses that use school attainment alone to represent adult cognitive skills are problematic.

In this paper, we examine the importance of pre-school, school, and post-school experiences in the production of both verbal and nonverbal cognitive skills of adults.^5^ We do this in Guatemala, a low-income context in which it is widely expected that greater cognitive skills will lead to increases in individuals’ options and welfare, and consequently advance economic development.

We utilize data that have been collected over 35 years (1969–2004) with sample members 25–42 years of age at the final survey and with substantial prospective and recall information on their development through the pre-school, school, and post-school years, as well as on a number of factors that conditioned these experiences. We investigate the effect of experiences during these three life-cycle stages on adult cognitive skills, incorporating the possibility that the experiences reflect behavioral choices in the presence of unobserved factors that might affect adult cognitive skills directly, as well as indirectly via their effects on the different life-cycle stage experiences. In addition to using plausibly exogenous instruments including an experimental intervention in childhood, identification is strengthened by the inclusion of the different life-cycle stages.

We find that (1) school attainment has a significant and substantial effect on adult verbal cognitive skills, but not on adult nonverbal cognitive skills; (2) pre-school and post-school experiences—represented, respectively, by the pre-school nutritional status of the individual and years of skilled post-school work experience— have substantial and significant effects on both of the adult cognitive skill measures; and (3) failure to control for the behavioral determinants of these experiences leads to substantially overestimating the importance of schooling while at the same time underestimating the importance of pre- and post-school experiences.

## 2 Conceptual framework

We estimate production functions for two types of adult cognitive skills, verbal (reading and vocabulary) and nonverbal. We posit that each skill is produced by the following: a vector of genetic and other observed and unobserved endowments related to learning capacities and motivations (**E_0_**); previous experiences (*E*_*i*_, *i* = 1, 2, 3 for the three life-cycle stages defined below); and a stochastic term (U) reflecting all other idiosyncratic, and assumed exogenous, experiences related to cognitive skill formation. Because of the nearly exclusive emphasis in the literature on schooling, and its plausibly important role in forming cognitive skills, we organize an individual’s experiences into three life-cycle stages:

Stage 1: pre-school (from conception through about age 6 years)
Stage 2: school (from age seven through about age 15 years),^6^
Stage 3: post-school (from about age 15 years old to age at the time of the final survey, i.e., 25–42 years).

The production functions for the two types of adult cognitive skills (*K*) are^7^

(1v) $$K_{3v}=K_{3v}^{P}(E_{1},E_{2},E_{3},E_{0r},U_{3v}),$$

and

(1n) $$K_{3n}=K_{3n}^{P}(E_{1},E_{2},E_{3},E_{0n},U_{3n})$$

The first subscript refers to the life-cycle stage, the second subscript refers to verbal cognitive skills (*v*) or nonverbal cognitive skills (*n*), and the superscript *p* indicates that the relation is a production function. While all variables except the left-side dependent variables and the disturbance terms are potentially vectors (indicated in bold), for tractability in the subsequent empirical analysis, we treat **E_1_, E_2_**, and **E_3_** as scalars *E*_1_, *E*_2_, and *E*_3_.

Our main research questions relate to the first derivatives (i.e., $K_{E1}^{p},K_{E2}^{p},K_{E3}^{p}$) of the general adult cognitive skills production functions. If $K_{E1}^{p}$ is significantly positive and *E*_1_ and *E*_2_ are positively correlated, for example, a specification that excludes *E*_1_ is likely to overestimate the effect of experiences during the school years (*E*_2_). Identification of relation (1v, 1n) is challenging, however, because the experiences for the three life-cycle stages on the right side all reflect previous behavioral choices.

To motivate our modeling of these life-cycle stage experiences and to elucidate the possible influences of the endowments on estimates that do not control for all three of them, we describe a stylized model in which the “dynasty” (first the parents, then the children themselves as they age into youth and adulthood) make decisions as if they were maximizing a welfare function (*W*) for each individual in adulthood that depends, *inter alia*, on the adult cognitive skills of that individual:

(2) $$W=W(K_{3v},K_{3n},\ldots U_{3v}).$$

For instance, *W* might represent consumption that is financed by resources generated by labor earnings that depend in part on cognitive skills. Welfare is maximized subject to the constraints at each life-cycle stage related to relevant current and expected production functions, family resources allocated to this individual, community characteristics (including community services and markets that affect household decisions), and stochastic factors.

Life-Cycle Stage 1 (pre-school years): The parents allocate resources to obtain the optimal *E*_1_ given a production function and the current community-determined options (e.g., availability of nutritional and health programs), expected future community-determined options (e.g., schooling options in the second life-cycle stage and labor market options in the third), the expected relation between *E*_1_ and *W*, and child-specific endowments. The *E*_1_ production function is

(3) $$E_{1}=E_{1}^{P}(N_{1},C_{1p},E_{0p},U_{1E}),$$

where **N_1_** is a vector of family determined inputs into the production of *E*_1_ (e.g., family provided nutrients), **C_1p_** is a vector of community inputs into the production of *E*_1_ (e.g., community-provided nutrients), **E_0p_** is the vector of child endowments that directly enter into the production of *E*_1_ (e.g., innate robustness), and *U*_1*E*_ is a stochastic disturbance term that directly affects the production of *E*_1_ (e.g., fluctuations in the infectious disease environment). Parents choose the inputs **N_1_** and, therefore, *E*_1_ to maximize the expected welfare *W*, given the following: a vector of parental family resources such as parental schooling and assets (**F_1_**); all relevant community characteristics for this life-cycle stage **C_1_** (which includes the community characteristics that directly affect the production of *E*_1_ through **C_1p_**, but also other community characteristics that may affect the household through other channels); all of the child endowments **E_0_** (which includes **E_0p_**, but also **E_0v_** and **E_0n_** that affect the decision to invest in *E*_1_ because the impact of *E*_1_ on *W* in general may depend on these other endowments); all the stochastic terms that affect outcomes in the first life-cycle stage of the child *U*_1_ (which includes *U*_1*E*_ but also other stochastic factors that affect the family during the first life-cycle stage for this child, for example, stochastic factors affecting the health of other siblings may influence the inputs devoted to this child); and, lastly, the expected values of these variables in the next two life-cycle stages ($F_{12}^{e},F_{13}^{e},C_{12}^{e},C_{13}^{e},U_{12}^{e},U_{13}^{e}$, where the superscript *e* indicates that the variable is an expected value, the first subscript refers to the life-cycle stage *at* which the expectations are held, and the second subscript refers to the stage for which the expectations are held) because the optimal decision for investing in *E*_1_ to maximize *W* depends in part on expectations of these variables over the next two life-cycle stages. The resulting relation is

(4) $$E_{1}=E_{1}^{d}(F_{1},C_{1},E_{0},U_{1},F_{12}^{e},F_{13}^{e},C_{12}^{e},C_{13}^{e},U_{12}^{e},U_{13}^{e}),$$

where the superscript *d* indicates that it is a reduced-form demand relation.

Life-Cycle Stage 2 (school years): The dynasty (initially the parents but with time increasingly the child) decides on the optimal *E*_2_ for the child/youth conditional on (a) the outcome of stage 1, *E*_1_, (b) life-cycle stage 2 family, community, and stochastic factors, and (c) the expected values of those factors for life-cycle stage 3. Using (4), this yields the reduced-form demand relation for *E*_2_:

(5) $$E_{2}=E_{2}^{d}(F_{1},C_{1},E_{0},F_{12}^{e},F_{13}^{e},C_{12}^{e},C_{13}^{e},F_{2},C_{2},F_{23}^{e},C_{23}^{e},U_{1},U_{2},U_{12}^{e},U_{13}^{e},U_{23}^{e}).$$

If the outcome of stage 1, *E*_1_, encompasses all the impacts of first life-cycle stage exogenous variables, ^8^ then the conditional (on *E*_1_) demand function for *E*_2_ is

(6) $$E_{2}=E_{2}^{c}(E_{1},F_{2},C_{2},F_{23}^{e},C_{23}^{e},U_{2},U_{23}^{e}),$$

where the superscript *c* refers to a conditional reduced-form demand relation. This relation allows for the possibility, for example, that the investment in *E*_2_ is dependent on *E*_1_.

Life-Cycle Stage 3 (post-school years): The dynasty (primarily the youth/young adult, but with possible input from parents) decides on the individual’s post-school experience *E*_3_ conditional on (a) the outcome of the first-stage *E*_1_, (b) the outcome of the second stage *E*_2_, and (c) the third stage family, community, and stochastic factors, yielding the reduced-form demand relation for *E*_3_:

(7) $$E_{3}=E_{3}^{d}(F_{1},C_{1},E_{0},F_{12}^{e},F_{13}^{e},C_{12}^{e},C_{13}^{e},F_{2},C_{2},F_{23}^{e},C_{23}^{e},F_{3},C_{3},U_{1},U_{2},U_{3},U_{12}^{e},U_{13}^{e},U_{23}^{e}).$$

The conditional demand function for *E*_3_ under the assumption that the outcome of stage 2, *E*_2_, encompasses all the impacts of second life-cycle stage predetermined variables (including *E*_1_) is

(8) $$E_{3}=E_{3}^{c}(E_{2},F_{3},C_{3},U_{3}).$$

This relation allows for the possibility, for example, that post-school experiences depend, *inter alia*, on previous school experiences and, through schooling, on preschool experiences.

This conceptual framework yields a number of important implications for the estimation of adult cognitive skills production functions.

1. *Omitted variable bias:* If the true relations (1v) and (1n) include all three life-cycle stages, excluding one or more of them may lead to substantial omitted variable biases. The endowments and the actual or expected values of the family, community, and stochastic factors for the three life-cycle stages are all on the right side of each of the reduced-form demand relations for each individual life-cycle stage (relations 4, 5, and 7) and, therefore, the three life-cycle experiences are likely correlated. The same conclusion can be drawn from the conditional demand functions (relations 6 and 8) in which school experience (*E*_2_) depends explicitly on pre-school experience (*E*_1_) and post- school experience (*E*_3_) depends explicitly on the school experience (*E*_2_), as well as indirectly on pre-school experience (*E*_1_). That the three life-cycle stage experiences are likely to be correlated is also intuitive. A priori, a child who lives in a household with better parental family background or who lives in a community with better health and educational services or employment opportunities, is likely not only to have more schooling but also better pre-and post-school experiences.
2. *Endogeneity bias:* Even when all three life-cycle stages are included, estimates of relations (1v) and (1n) that do not attempt to control for their behavioral determinants are also likely to be biased. Moreover, the signs of such biases are ambiguous. For instance, “ability bias” is consistent with positive correlation between *E*_2_ (e.g., if measured with school attainment) and the innate ability component of **E_0_**, suggesting a likely upward bias on *E*_2_ in ordinary least squares (OLS) estimates. However, if the summary measure of pre-school experience is some variable such as child nutritional status (see Sect. 3 below), and if ability and physical endowments that influence nutritional status are negatively correlated as suggested by Behrman and Rosenzweig (2002), estimates for child nutritional status (*E*_1_) in relations (1v) and (1n) may be negatively biased while at the same time estimates for school attainment (*E*_2_) are positively biased.^9^
3. *The same potential instruments identify all three life-cycle stage experiences:* The reduced-form demand relations for the three life-cycle stage experiences in relations (4), (5), and (7) suggest potential instruments that could be used to identify the different life-cycle stage experiences in the adult cognitive skills production functions in (1v) and (1n). Although some instruments may seem likely to have first-order effects on particular life-cycle stages (e.g., pre-school nutrition programs on *E*_1_, school characteristics on *E*_2_, labor market characteristics on *E*_3_), the three reduced-form demand relations make clear that the same actual or expected values of the family, community, and stochastic factors can all influence each of the three life-cycle stages. Consequently, our approach is oriented around two-stage least squares rather than an instrumental variable approach in which different instruments are excluded from the various first-stage predictions. By capturing all three life-cycle stages, other pathways through which the instruments might influence cognitive skills are directly included.
4. *Instruments must be uncorrelated with the disturbance term, which includes unobserved endowments:* The reduced-form demand relations indicate the potential set of instruments, but not all of the right side variables in those relations are likely to be valid instruments in the sense of being uncorrelated with the disturbance terms in relations (1v) and (1n). For example, previous studies make clear that there can be intergenerational correlations of endowments through genetics and probably other means (Behrman and Rosenzweig 1999, 2002, 2005; Stein et al. 2003). To limit this possible correlation, we do not include variables such as parental school attainment or parental wealth as instruments in our preferred models.

## 3 Data

Data demands are considerable for estimating these adult cognitive skills production functions. We use longitudinal data from Guatemala collected over a 35-year period with anthropometric, socioeconomic, and adult cognitive skills measures, shocks from an experimental intervention and a major earthquake, and other market and policy changes.

### 3.1 The INCAP experimental nutritional intervention

In 1969, the Institute of Nutrition of Central America and Panama (INCAP), based on Guatemala, began a nutritional supplementation trial (Martorell et al. 1995a). After screening approximately 300 communities in eastern Guatemala, two sets of community pairs (one pair with about 500 residents each and the other with about 900 residents each) were selected purposively for the trial. Two of the communities, one from within each pair matched on population size, were assigned randomly to receive as a dietary supplement a high protein-energy supplement, *atole*. The other two communities received an alternative no-protein, low-energy supplement, *fresco*.

Collection of the data used in this paper began with the supplementation trial in these four communities. From February 1969 to February 1977, INCAP implemented the nutritional supplementation program, together with data collection on child growth and development. The survey associated with the intervention focused on all children under 7 years of age, including children born in the study area during those years. Detailed data collection for individual children ceased when they reached 7 years of age or when the study ended, whichever came first. The children included in the 1969–1977 longitudinal survey were thus born between 1962 and 1977, and consequently the length and timing of exposure to the interventions for specific children depended on their individual birth dates. For example, only children born after early 1969 and before February 1974 were exposed to an intervention for all of the time from birth to 36 months of age, posited in the nutritional literature (Sect. 3.2) to be a critical time period for child growth. *Atole* and *fresco* were distributed in each community in centrally located feeding centers and were available daily, on a voluntary basis, to all members of the community, regardless of their age or participation in the survey components of the research, during times that were convenient to mothers and children, but that did not interfere with usual meal times.

Because we use children’s differential exposure to the availability of the nutritional supplements as instruments to estimate first-stage relations (4), (5), and (7), that are then excluded from relation (1v, 1n), it is important to establish that the two interventions resulted in differential consumption of calories, protein, and other nutrients. Approximately, 70 % of children between the ages of 0–36 months consumed at least some *atole*, with no difference between boys and girls. Similar overall participation rates were observed in *fresco* communities. Averaging over all children in the *atole* communities (regardless of their levels of voluntary participation), children 6–12 months consumed approximately 70 kcal of *atole* supplement per day; children 12–24 months, 90 kcal; and children 24–36 months, 120 kcal. Children in the *fresco* communities, however, consumed only 20 kcal of *fresco* supplement per day between the ages of 6–24 months, rising to approximately 30 kcal by the age of 36 months (Schroeder et al. 1992; Islam and Hoddinott 2009).

In 2002–2004, a team of investigators, including most of the authors of this paper, undertook a follow-up survey targeted to all subjects in the original survey.^10^ At the time of the final survey, sample members ranged from 25 to 42 years of age. Of the 2,392 individuals in the original 1969–1977 sample by the time of the 2002–4 HCS, 1,855 (78 %) were alive and known to be living in Guatemala (11 % had died—the majority due to infectious diseases in early childhood, 7 % had migrated abroad, and 4 % were not traceable). Of these 1,855, 60 % lived in the original communities, 8 % lived in nearby communities, 23 % lived in or near Guatemala City, and 9 % lived elsewhere in Guatemala. Of the 1,855 traceable sample members living in Guatemala, 1,571 (85 %) completed at least one interview during the 2002–2004 survey (Grajeda et al. 2005). This study includes the 1,448 respondents (46 % male) interviewed in 2002–2004 for whom the two measures of adult cognitive skills we use in the main analyses were available (Sect. 3.2). They comprise 78 % of the 1,855 individuals who were alive and known to be living in Guatemala and 61 % of the original sample. Measured from 1977 to 2002, the latter figure indicates an annual attrition rate of approximately 2 %, low when compared to shorter-term longitudinal surveys in developing countries (Alderman et al. 2001) or to long-term longitudinal surveys in developed countries (Fitzgerald et al. 1998).^11^ Nevertheless, nearly 40 % represents substantial attrition and, therefore, we assess potential attrition biases (Sect. 4.2.4).

### 3.2 Central variables for the analysis

Table 1 presents summary statistics for the 1,448 individuals used in the main analyses.

#### 3.2.1 Dependent variables: adult cognitive skills (K)

1. Verbal cognitive skills: The vocabulary (approximately fourth-grade equivalent) and reading comprehension (approximately 3rd grade equivalent) modules of the Inter-American Reading and Comprehension Tests (Manuel 1967) were administered to the 1,197 individuals (83 % of the sample of 1,448) who reported completing six or more grades of school or who passed a pre-literacy screen.^12^ The vocabulary portion had 45 questions and reading comprehension had 40 questions, yielding a maximum possible score of 85 points. The distribution of test scores (for those who took the test) appears to be symmetric and approximately normal. The 17 % of the sample who did not pass the pre-literacy screen were assigned a value of zero for the reading comprehension tests. Including those we scored at zero, the mean score is 36.1 with a standard deviation (SD) of 22.3, indicating substantial sample variation (Table 1). Women (mean 34.4, SD 21.9) score significantly lower than men (mean 37.9, SD 22.7). These tests had adequate test–retest reliability (correlation coefficients of 0.87 and 0.85 for vocabulary and reading) in previous studies in this population when the same subjects were adolescents and young adults (Pollitt et al. 1993). We use the combined vocabulary and reading comprehension score (hereafter, RCS) as our measure of verbal cognitive skills. Because of the nature of the distribution of the test scores, in the empirical analysis, we explore the robustness of our results to changes in the specification of these test scores, including directly incorporating the pre-literacy test score (Sect. 4.2.3).
2. Nonverbal cognitive skills: All respondents were administered Raven’s Progressive Matrices (Raven et al. 1984), a widely used nonverbal measure of interpretative cognitive skills,^13^ that consists of a set of shapes and patterns, with the respondent asked to supply the “missing piece” from a set of six choices. The Raven thus measures different aspects of cognition than the verbal skills described above, although they are highly correlated (0.57) and may have some components of general cognitive skills in common. We used the first three of the five scales (with 12 questions each, for a maximum possible score of 36) because pilot data suggested and subsequent survey data confirmed that few respondents were able to progress beyond the third scale. As with the test of reading and vocabulary, there is considerable sample variation; similar to the distribution of verbal skills, the distribution of these test scores also appears to be symmetric and approximately normal, with a mean of 17.7 points (SD 6.1). Women (mean 16.3, SD 5.4) score significantly lower than men (mean 19.4, SD 6.5). The Raven’s test also exhibited adequate test–retest reliability (correlation of 0.87) in this population in the past (Pollitt et al. 1993). We use the Raven’s test scores as our measure of nonverbal cognitive skills.

#### 3.2.2 Life-cycle stage experiences (E_1_, E_2_, E_3_)

We assume relation (1v, 1n) is linear and include one commonly used indicator each for the first two life-cycle stages.^14^ For the third stage, we consider two different indicators of potentially relevant post-school experiences. Technically, then, we assume the indicators we use are sufficient statistics for each stage, though practically they are probably better interpreted as being among the most important experiences related to adult cognitive skill development.

##### Pre-school experience

There is substantial emphasis in the literature on the importance of nutrition—reflecting nutrient intakes and exposure to infections—as measured by height in early childhood development. We use pre-school child height-for-age Z scores (HAZ) at age six,^15^ a widely accepted indicator of childhood long-run nutritional status, to calculate our indicator for pre-school experience. There is no difference between average female and male HAZ.

##### School experience

We use completed school attainment (highest grade completed) as our indicator of experiences related to cognitive skill formation during this life-cycle stage. The mean is 4.7 grades (SD 3.5). Women (mean 4.3, SD 3.3) average significantly fewer completed grades than do men (mean 5.2, SD 3.6). The distribution has a local mode at six grades (29 % of the individuals), completion of primary school. There are also secondary modes at zero grades (14 %) and three grades (11 %). School attainment is significantly correlated with the indicators for the pre-school experience (correlation of 0.16) and post-school experiences described below (skilled job tenure, 0.13; whether living in Guatemala City, 0.28), suggesting that if relation (1v, 1n) is the true relation but only school attainment is included in the estimation, the estimate of the effect of the latter is likely to be biased.

##### Post-school experience

We consider two measures for this third life-cycle stage.

- *Tenure in skilled jobs*: We use the duration in years of continuous work experience prior to the 2002–2004 survey in skilled jobs^16^ as our first indicator of post-school experience that may contribute to adult cognitive skills. The survey does not permit the calculation of total experience in all jobs since individuals left school, but does capture tenure in all jobs held in 1998 and in 2002–2004.^17^ Such experience is likely to strengthen cognitive skills via (1) learning-by-doing through problem solving; (2) furthering skills learned in school by using them in real world applications; and (3) exposing individuals to a wider environment, through interactions with coworkers and customers. The mean number of years of tenure in a skilled job is only 2.8, but with substantial variation (SD 4.8). Two-thirds of the individuals were not in skilled jobs in 1998 or 2002–2004 and, therefore, have reported tenure of zero years. Among the 530 sample members who had at least 1 year of tenure in a skilled job, the mean is 7.8 years (SD 5.0). There are substantial and significant differences for tenure in skilled jobs by gender, with 58 % of men having such experience (mean 8.1 years, SD 5.0), but only 18 % of women (mean 6.9 years, SD 5.0).
- *Migration to Guatemala City*: Living in Guatemala City, rather than in one of the original sample communities or elsewhere in Guatemala, might alter experiences related to cognitive skill formation through work, but also via shopping, entertainment, transportation, or many other aspects of life in a major city. Seventeen percent of the sample lived in Guatemala City at the time of the 2002–2004 survey, 18 % of women and 15 % of men. The mean of the estimated number of years that these individuals had been away from their origin community is 3.9 years (SD 6.6), with no significant difference by gender, even though men have been in the city for, on average, one additional year (i.e., on average, women had spent an additional year in their original community).

The two indicators have low correlation (0.04), underscoring the possibility that they might capture different dimensions of post-school experiences.

#### 3.2.3 Initial conditions (**E_0_, F_0_**)

##### *Genetic and other endowments and other individual characteristics* (**E_0_**)

We do not have direct observations on genetic endowments beyond the gender of the individual, which we control for in our first-stage estimates. Other individual characteristics that we observe include age at the time of interview (which we include in both the first and second stages) and whether the individual was a twin, which may have longer-run implications through the human capital investments across the life-cycle (Behrman and Rosenzweig 2004). If the instruments that are only in the first stage are uncorrelated with individual-level endowments, however, failure to include direct measures of the endowments will not lead to biased estimation of relation (1v, 1n), since the predicted life-cycle stage experience measures will be orthogonal to endowments in the error term.

##### *Parental wealth and school attainment* (**F_0_**)

In some specifications, although not our preferred ones because of their likely association with unmeasured endowments through intergenerational transmission, we also consider the role of parental characteristics in the production of cognitive skills.

#### 3.2.4 Observed events and shocks (***C***)

##### Experimental nutritional shocks

One observed shock is the nutritional intervention underlying the original study. The *atole* intervention has been shown to have improved child growth (Martorell et al. 1995b). We construct two intent-to-treat measures, based on the community and date of birth of each individual and the dates of operation of the interventions. For each individual, we calculate whether they were exposed to either intervention for the entire period from 0–36 months of age. A potential exposure to the *atole* intervention (relative to *fresco*) is then calculated by multiplying this cohort measure by a dummy indicator of whether or not the child lived in one of the two *atole* communities.

##### Natural, market, or policy events

Guatemala suffered a major earthquake in 1976 and we incorporate a dummy variable for whether an individual was born in the 2 years prior to it because the economic shock due to the earthquake may have been particularly important for infants and very young children. We also include community-level variables that relate as closely as possible to the timing of key schooling- and labor market-related decisions in each individual’s development. Using information reported in earlier work about infrastructure, markets, and services in the communities, complemented with a retrospective study in 2002, we construct the student–teacher ratio and an indicator of whether a lower secondary school (grades 7–9) was available, both measured in the community in which the individual lived when the individual was 7 years old. To capture changes in local market conditions, we construct a variable indicating the logarithm of the (national) salary in manufacturing when the individual was 18 years old and likely to be in their early working years. Thus, while reflecting community-level characteristics (except for the manufacturing salaries), these variables vary by single-year age cohorts within each community, as well as across communities. This is preferable to the more common approach of using measures of such factors at a given time for a population with different ages at that point in time, since our indicators more closely relate to periods in individuals’ lives when critical decisions (e.g., starting school) were being made. We also include an additional individual-specific shock—whether the individuals’ father or mother had died (prematurely and possibly accidentally) by the time they were 18, which relates to a critical juncture for entering the labor force.^18^

## 4 Results

In Sect. 4.1, we summarize our main estimates, using indicators of experiences during each of the three life-cycle stages, and endogenizing using a two-step instrumental variable feasible generalized methods of moments (IV-GMM) estimator (Baum et al. 2007). In Sect. 4.2, we consider how robust estimates are to gender differences, variations in the instrumental variables used, alternative representations of verbal cognitive skills, and further controls for attrition. We allow for clustering at the birth-year-community cohort level in the calculation of the standard errors to control for potential serial correlation among children born in the same community in the same year; this yields 64 clusters.^19^

### 4.1 Basic specification of the adult cognitive skills production functions

Our identification strategy has three components. First, using the framework developed in Sect. 2, we select plausibly exogenous characteristics from the individuals’ backgrounds and communities to predict the three E_i_ life-cycle stage outcomes. These include community-level market and policy shock variables derived from the detailed community histories and the intent-to-treat exposure variables derived from the original nutritional supplementation intervention, as well as an indicator for having been born just prior to the 1976 earthquake. Second, we include *all three* of these life-cycle stages in our main specification, which means that the possibility that the instruments have direct effects that do not operate through the included measured experiences in the second stage of the model is substantially mitigated, especially in comparison to a model which excluded one or more of those experiences. For example, in our main specifications, the presence of a lower secondary school at age 7 years would be an invalid instrument only if it had a direct effect above and beyond its (linear) effect operating through each of the three *E_i_*, pre-school nutrition (through expectations), school attainment, *and* post-school skilled job tenure. Third, we carry out a range of diagnostic tests to assess the strength and validity of the instruments, as well as consider alternative instrument sets and estimation procedures.

#### 4.1.1 First-stage estimates

The first-stage estimates have a number of individually significant point estimates consistent with our hypotheses about how the instruments affect each of the life-cycle stages (Table 2). Being a twin or being born within the 2 years prior to the 1976 earthquake reduces the HAZ at age six, whereas exposure to the *atole* intervention in the first 3 years (relative to *fresco*) increases it. Being male, an individual living in a community with a lower secondary school at age seven, or exposed to higher salaries in manufacturing at age 18, all lead to higher school attainment. A notable period of rising wages occurred in the late 1970s with reconstruction after the 1976 earthquake and in the late 1980s and early 1990s, associated with a building boom in Guatemala City and higher school attainment was one of the criteria used in hiring in manufacturing and other industries. For example, completed third grade was a requirement for even the lowest level jobs available in a local cement factory that opened in the 1980s. The loss of a parent before age 18 years and higher student–teacher ratios, on the other hand, are associated with lower school attainment. Men have significantly more tenure in skilled jobs. Exposure to *atole* when 0–36 months, lower secondary school available at age seven, and higher manufacturing wages at age 18 are linked to lower tenure in skilled jobs, consistent with individuals being more likely to go into low-skill manufacturing. The probability of migration to Guatemala City increases with lower secondary school being available at age 7 years and with higher manufacturing wages at age 18 years.

Table 3 presents four sets of estimates related to the linear version of the adult cognitive skills production function in relation (1v, 1n) for verbal cognitive skills in panel A and for nonverbal skills in panel B. Both are represented as Z scores (i.e., internally standardized in terms of their sample means and SDs). Three of the sets of estimates sequentially add experience in the chronological order of the three life-cycle stage experiences as follows: only pre-school experience (HAZ at age six) (Set 1A); pre-school experience and school-years experience (school attainment) (Set 2); and all three life-cycle stage experiences—HAZ at age six, school attainment, and skilled job tenure (Set 3). These alternative specifications show how the coefficient estimates of the earlier life-cycle stage experiences change as later experiences are incorporated. To permit comparisons with previous literature that typically does not included pre- or post-school experiences, we also present estimates that include only school attainment (Set 1B). For each set, we present both OLS and IV-GMM estimates (Baum et al. 2007; Hayashi 2000), using the instruments indicated in Table 2. For the final specification with all three life-cycle experiences, we also present IV limited information maximum likelihood (LIML) results using the same set of excluded instruments.

#### 4.1.2 IV diagnostics

For the full specifications with all three life-cycle experiences (Set 3), the first- and second-stage diagnostics are satisfactory for both types of skills; the instruments we utilize have reasonable power and we fail to reject the overidentification tests. The *F* tests on excluded instruments range from 7.3 to 26.3 (Table 2) (Bound et al. 1995) and the Kleibergen–Paap (KP) Wald *F* statistics for weak instruments are significant at a 5 % significance level (Table 3, bottom panel).^20^ The Hansen J (HJ) statistics for overidentification indicate that the first-stage instruments are not correlated with the second-stage disturbance term.^21^ Finally, the Hausman tests indicate that the IV-GMM estimates differ significantly from OLS for both verbal and nonverbal skills (Hayashi 2000; StataCorp. 2011). By contrast, in four of the six specifications in which one or two of the life-cycle stage experiences are excluded from the specification of relation (1v, 1n), the HJ tests indicate that there is a specification problem, consistent with concerns that the excluded instruments are correlated with the omitted life-cycle experience which belongs in the model.

#### 4.1.3 Pre-school experience (E_1_)—HAZ at age six

The OLS associations between HAZ at age 6 years and both cognitive skills measures, conditional on a quadratic in age, are positive, significant, and fairly substantial, 0.20 SD for verbal skills and 0.19 SD for nonverbal skills (Table 3, first column in top two panels). If school attainment and skilled job tenure are included and along with HAZ at age 6 years are treated as behaviorally determined (IV-GMM estimates in the second to last column), the estimated effect of HAZ at age 6 years is similar, though now only marginally significant (*p* = 0.08) for verbal skills, but much larger and significant for nonverbal skills (0.33 SD). Therefore, our preferred estimates with all three life-cycle stages treated as endogenous indicate a substantial gain from higher HAZ at age 6 years in terms of both verbal and nonverbal skills more than 20 years later, controlling for both school- and post-school experiences and for the endogenous determination of all three of these life-cycle experiences. The alternative specifications for nonverbal skills indicate that the coefficient estimate for HAZ at age 6 years and for skilled job tenure increase markedly when moving from OLS to IV-GMM estimates in the full specification. This pattern is consistent with a negative correlation between the unobserved endowment components positively related to skill development and school attainment and the endowment components positively related to biological development (Behrman and Rosenzweig 2004), and skilled job tenure. Without controls for the endogenous determination of the life-cycle experiences, the effects of HAZ at age 6 years and of skilled job tenure on adult nonverbal skills appear to be substantially underestimated.^22^

#### 4.1.4 School-age experience (E_2_)—school attainment

If only school attainment is included using OLS, the estimated associations indicate increases of 0.22 SD of verbal skills and 0.15 SD of nonverbal skills for each additional grade attained. These are fairly substantial effects, implying, for example, increases of approximately 0.75 SD in verbal skills and of 0.50 SD in nonverbal skills for a 1 SD increase in school attainment. For verbal skills, however, this estimated effect is halved if IV estimates are used for school attainment, whether or not the other life-cycle experiences are included. Our preferred estimate for the school attainment coefficient from the IV-GMM estimates, with all three life-cycle experiences included, is 0.09 SD in verbal skills for every additional grade, or approximately 0.30 SD in verbal skills for a 1 SD increase in school attainment. While still an important effect, it is only about half of estimated effect using OLS. For nonverbal skills, the combination of including post-school experience and treating all three life-cycle experiences as behaviorally determined results in a decline in the estimated coefficient on school attainment to 0.04 SD for every additional grade, and it is no longer statistically significant. This is consistent with the claim that the Raven’s tests are not affected by schooling per se, but instead may reflect attributes that themselves affect schooling (Schweizer et al. 2007).

#### 4.1.5 Post-school-age experience (E_3_)—skilled job tenure

For verbal skills, we find a small but significant impact on the IV-GMM estimates of skilled job tenure of 0.03 SD for every year of such tenure. For nonverbal skills, we find a significant impact of skilled job tenure that implies an increase of 0.14 SD for every additional year of skilled job tenure. This is a fairly substantial effect, implying an increase of approximately 0.67 SD in nonverbal skills for a 1 SD increase in skilled job tenure.^23^ In Sect. 3.2, we discussed the possibility that migration to Guatemala City, the capital, also might provide experiences that could improve cognitive skills. We examined this possibility by including an indicator for migration to the capital, in addition to the other three experience measures, to the specifications shown in the final columns of Table 3, treating migration to the city as endogenous. For both verbal and nonverbal skills, the results for the other life-cycle stages are unchanged, and the coefficient on migration, while large, is insignificant (results not shown). While the F statistic for the excluded instruments on the migration indicator was 7.8 (Table 2), the KP statistic (3.4) indicates that the instruments as a set are weak when we endogenize all four of these measures.

The final column in Table 3 presents the model estimated by LIML, using the same instrumental variables as in the IV-GMM specifications. Results are similar, although the estimates of the effect of HAZ at age six are less precise and no longer marginally significant for verbal skills but still marginally significant (*p* = 0.08) for nonverbal skills.^24^ Conditional on the other variables, both types of skills decline slightly with age.

### 4.2 Robustness considerations

#### 4.2.1 Gender differences

Adult men in the sample score significantly higher on the tests of cognitive skills than women. This raises the question to what extent the patterns above reflect differences in the cognitive skills production functions or life-cycle stage experiences between men and women. One simple alternative is to incorporate gender directly into the production function, as we do with age.^25^ Specifically, we include a male dummy variable in the second-stage estimated relations.^26^ When we do so, the results with respect to the influence of the three life-cycle stages on verbal and nonverbal skills are similar to those in Table 3. The first-stage diagnostics, however, indicate that the instruments are weak, with a KP Wald *F* statistic of 3.2, so we do not report them. Consequently, while there is no evidence of substantial differences in the relationships for men versus women, such differences are hard to identify well in these data.

#### 4.2.2 Alternative instrumental variables

A second concern is that the HJ statistic may have low power and, despite the multiple life-cycle stages in the model, some of the instruments are invalid, i.e., correlated with the error term. For either outcome, however, when we include parental characteristics^27^ in the set of excluded instruments, the HJ statistic rejects (*p* < 0.003) the null hypothesis that the overidentifying restrictions are valid. This suggests that the HJ does have sufficient power to detect invalid instruments, giving us greater confidence in the main results that exclude these parental characteristics from the first-stage instruments.

#### 4.2.3 Alternative formulations of verbal skills

Due to the literacy screen (Sect. 3), the vocabulary and reading comprehension scores (RCS) on which verbal skills were based had a mass point at zero. In Table 4, we present alternative estimates (using the same instrument set as our preferred estimates in Table 3) that address this concentration of scores: (1) instrumental variables Tobit estimates using the verbal scores that account for the lower bound and mass point at zero; (2) IV-GMM estimates using RCS + pre-literacy test scores; and (3) IV-GMM estimates of the quartile of the RCS score.^28^ The significance of HAZ is not robust to all three alternative specifications, but the findings regarding school attainment and skilled job tenure hold, with the latter significant at the 5 % level in the quartile specification but only at the 10 % level in the other two specifications. We conclude that these alternative representations do not alter substantially our qualitative findings or change our interpretations in Sect. 4.1.

#### 4.2.4 Attrition

Despite the considerable effort and success in tracing and reinterviewing participants from the original sample, attrition in our sample is substantial and associated with a number of initial conditions (Grajeda et al. 2005). What is of ultimate concern in this analysis, however, is not the level of attrition, but whether the attrition invalidates the inferences we make using these data. For example, does excluding migrants who were not located and who may have different characteristics lead to systematic bias of the estimates presented here?

To explore these concerns, we implement the correction procedure for selective attrition on observed characteristics outlined in Fitzgerald et al. (1998). We first estimate an attrition probit conditioning on all the right side variables (including instruments) considered in the main models, as well as an additional set of (endogenous) variables potentially associated with attrition, for all original sample members (*N* = 2,392). The latter variables include factors that reflect family structure in previous years, as these are likely to be associated with migration status: whether an individual lived with both their parents in 1975 and in 1987. During the fieldwork, locating sample members was facilitated by having access to other family members from whom the field team could gather information. Therefore, we also include variables that capture this feature of the success of tracking migrants: whether the parents were alive in 2002, whether they lived in the original community, whether a sibling of the sample member had been interviewed in the 2002–2004 follow-up survey, and the number of siblings in the family in the original sample. While we do not have adjustments to correct for selection on unobservable characteristics, by including a number of endogenous observables indicated above, which are likely to be correlated with unobservables, we expect that we are reducing the scope for attrition bias due to components of unobservables that are correlated with the included observed variables, as well.

Nearly all of the factors described above are significant and highly associated with attrition (Appendix Table 6). Following Fitzgerald et al. (1998), we construct weights that give greater weight to observations in the sample re-interviewed in 2002–2004 that had lower predicted probabilities of having been re-interviewed. Table 5 shows that application of these weights affects only slightly the results and that the central patterns of the coefficient estimates remain similar to those in our main estimates in the final column of Table 3, with the exception that the previously marginally significant HAZ score is now insignificant (*p* = 0.16). As found in other contexts with high attrition, we do not find evidence that the results are biased by attrition based on tests that use characteristics measured at baseline and during final round field work (Alderman et al. 2001; Fitzgerald et al. 1998).

## 5 Conclusions

Most empirical investigations of the effects of cognitive skills assume that they are produced by schooling, and that schooling is exogenous. We argue that such approaches are likely to lead to incorrect inferences not only about the effect of school attainment, but also about the potential importance of pre-school and post-school experiences on adults cognitive skills. To explore this, we draw on longitudinal data set to estimate production functions for adult verbal and nonverbal cognitive skills as dependent on behaviorally determined pre-school, school, and post-school experiences. We present a basic specification as well as a range of tests of how robust the basic specification is. While some of the robustness tests suggest qualifications, overall they support the following results:

1. School attainment has a significant and substantial effect on adult verbal skills, but not on adult nonverbal cognitive skills.
2. Pre-school and post-school experiences have substantial positive significant effects on adult cognitive skills.^29^ Pre-school experiences increase HAZ at age 6 years substantially and significantly increase verbal and nonverbal cognitive skills, even after controlling for school attainment and post-school skilled job tenure. The effect of HAZ on verbal skills, however, is less robust to changes in the methodology. Post-school tenure in skilled jobs has a significant positive effect on both verbal and nonverbal cognitive skills.
3. Estimates that do not account for the endogenous determination of these three life-cycle experiences are misleading. Estimates of the effect of schooling on adult cognitive skills that do not account for school attainment being behaviorally determined are biased upward substantially for adult verbal cognitive skills and make the impact on adult nonverbal cognitive skills appear highly positively significant rather than insignificant. Treating pre- and post-school experiences as statistically predetermined substantially underestimates their impacts. This contrasts with the upward bias for schooling, suggesting that the underlying physical and job-related components of genetic endowments are negatively correlated with those for cognitive skills.

While these results are of considerable interest in their own right, they also relate to four broader literatures.

First, in both developed and developing countries, there is growing interest in investing in disadvantaged children at an early stage in life. Drawing on a wide body of evidence from economics, psychology, and neuroscience, for example, Heckman (Heckman 2006a, b) argues that returns to such investments are much higher than those made later in life. However, the empirical base for these arguments is not as deep as would be desirable. For example, there are few studies that follow disadvantaged individuals over long periods of time. Our study adds to this literature by demonstrating that having relatively good nutritional status as preschoolers results in greater cognitive skills decades later as adults.

Second, a growing body of evidence suggests that, across a wide range of countries, scores on certain measures of cognitive skills or ability—including the Raven’s Progressive Matrices used in our study—are increasing over time. Referred to as the Flynn effect (Flynn 1987), Dickens and Flynn (2001) posit several pathways by which changes in environmental or behavioral factors, rather than in genetic factors, could cause scores on cognitive tests to increase over time. One of these is improved childhood nutrition. A second is increased cognitive complexity in the workplace. Our results for the impact of early childhood nutrition and for years of experience in skilled jobs—both treated as endogenous—provide direct evidence of the importance of these factors in shaping dimensions of cognitive skills that are consistent with the environmental and behavioral factors hypothesized to underlie the Flynn effect.

Third, a relatively small literature attempts to use what are interpreted to be direct measures of innate ability to examine whether human capital is associated with greater productivity as opposed to being mainly a signaling device (Boissiere et al. 1985; Alderman et al. 1996). Implicit in such approaches is the assumption that causality runs from cognitive abilities to productivities. But if more productive, higher remunerated work is also more complex, and if undertaking complex work improves cognitive skills, causality (also) runs the other way. We find evidence of this latter relationship. Therefore, our findings imply that studies that regress productivity on contemporaneous measures of cognitive abilities are flawed if they fail to take the endogeneity of cognitive skills into account.

And, fourth, there is considerable debate over the impact of human capital on income levels and growth. In this literature, aggregate country level estimates are presented in which human capital is typically represented by converting schooling enrollment rates into estimates of the stock of schooling (Nehru et al. 1995) or by school attainment for those older than 25 (Barro and Lee 1993). In doing so, these approaches assume that individuals do not accumulate additional human capital after completing schooling or after a certain age, nor does their human capital depreciate. Our results are at odds with this common assumption—we find that adult cognitive skills increase with experience in higher-skilled jobs (treated endoge-nously) but decline with age for our sample of 25–42 year olds. At the cross-country level, this implies that widely used representations of knowledge are problematic— they likely overstate human capital in slow-growing or traditional/subsistence economies and understate it in faster growing, modernizing economies. These biases are likely to result in underestimates of the importance of human capital in economic growth processes.

## Figures and Tables

**Table 1:** Summary statistics: mean and standard deviations (*N* = 1,448)

	All	Women	Men
**K_3_**: Cognitive skills			
Reading comprehension score (RCS) or verbal skills	36.05 (22.3)	34.41 (21.9)	37.95* (22.7)
RCS + pre-literacy test scores (RCS + prelit)	66.93 (29.7)	64.74 (29.9)	69.45* (29.2)
Raven score or nonverbal skills	17.70 (6.1)	16.26 (5.4)	19.36* (6.5)
E: Life-cycle stage experiences			
E_1_: HAZ at age six (1,268 non-missing cases)	−2.22 (0.96)	−2.20 (0.95)	−2.30 (0.97)
**E_2_**: School attainment (grades)	4.69 (3.5)	4.29 (3.3)	5.15* (3.6)
**E_3_**: Skilled job tenure (years)	2.85 (4.8)	1.27 (3.4)	4.67* (5.5)
Migrant to Guatemala City in 2002–2004 (=1)	0.17	0.18	0.15
**I_0_**: Individual characteristics			
Age at interview in 2002–2004 (years)	32.33 (4.2)	32.42 (4.3)	32.23 (4.2)
Age at interview in 2002–2004 squared (years )	1,063.2 (277.8)	1,069.5 (281.6)	1,056.0 (273.4)
Instruments			
**E_0_**: Genetic endowment			
Male (=1)	0.46	0.00	1.00
Twin (=1)	0.02	0.02	0.01
**F_0_**: Household and parental characteristics			
Household wealth index z score [1,330 non-missing cases]	−3.10 (0.92)	−3.10 (0.91)	−3.09 (0.93)
Mother’s school attainment (grades) [1,427 non-missing cases]	1.36 (1.7)	1.25 (1.6)	1.48* (1.8)
Father’s school attainment (grades) [1,344 non-missing cases]	1.71 (2.1)	1.69 (2.0)	1.77 (2.3)
**ΔC**: Natural, market or policy shocks			
Mother or father had died before child age 18 (= 1)	0.08	0.08	0.09
Student-teacher ratio at child age 7	39.93 (9.0)	40.26 (9.4)	39.55 (8.5)
Lower secondary school available at child age 7 (= 1)	0.21	0.21	0.21
Child born during the two years prior to 1976 earthquake (=1)	0.14	0.15	0.13
Ln salary in manufacturing industry at child age 18	1.60 (0.5)	1.58 (0.5)	1.61 (0.5)
Intent-to-treat nutritional intervention dummies			
Exposure 00–36 months (=1)	0.39	0.37	0.42
Exposure 00–36 months × *atole* (=1)	0.21	0.21	0.22
Observations	1,448	775	673

Standard deviations are shown in parentheses

* Difference between women and men based on two-sided *t* test with unequal variances is significant at a 5 % or lower

**Table 2:** First-stage estimates of life-cycle stage experiences (JV = 1,448)

	E1 HAZ at age 6	E2 Grades of schooling	E3a Skilled job tenure (years)	E3b Migrant to Guatemala City
**I_0_**: Individual characteristics				
Age at interview in 2002–2004	−0.098 (0.173)	1.284 (0.835)	0.203 (0.676)	**0.478** (0.092)
Age at interview in 2002–2004 squared	0.002 (0.002)	−0.015 (0.011)	−0.007 (0.009)	−**0.005** (0.001)
**E_0_**: Genetic endowment				
Male	−0.091 (0.049)	**0.802** (0.183)	**3.419** (0.250)	−**0.040** (0.018)
Twin	−**1.171** (0.273)	−0.311 (0.716)	−0.645 (0.666)	−0.041 (0.087)
**ΔC**: Natural, market or policy shocks				
Mother or father had died before child age 18	0.048 (0.083)	−**0.941** (0.263)	−0.400 (0.532)	−0.020 (0.034)
Student-teacher ratio at age 7	0.003 (0.002)	−.**034** (0.014)	−0.007 (0.013)	−0.001 (0.002)
Lower secondary school available at age 7	−0.038 (0.054)	**1.527** (0.251)	−**0.599** (0.290)	**0.102** (0.027)
Born in the two years prior to 1976 earthquake	−**0.221** (0.087)	−0.403 (0.418)	−0.251 (0.279)	0.012 (0.042)
Ln salary in manufacturing industry at child age 18	0.640 (0.332)	**3.132** (1.442)	−**3.503** (1.276)	**1.174** (0.176)
Intent-to-treat nutritional intervention				
Exposure 00–36 months	−**0.268** (0.068)	0.232 (0.391)	0.053 (0.387)	0.060 (0.045)
Exposure 00–36 months × *Atole*	**0.271** (0.075)	−0.348 (0.308)	−**0.767** (0.368)	0.017 (0.040)
Constant	−2.225 (3.804)	−24.698 (17.535)	8.694 (14.370)	−**11.742** (2.000)
*F* statistic on instruments excluded in second stage	7.3	15.4	26.3	7.8
[*p* value]	[<0.01]	[<0.01]	[<0.01]	[<0.01]
*F* statistic overall regression	15.6	17.6	24.8	7.2
[*p* value]	[<0.01]	[<0.01]	[<0.01]	[<0.01]

OLS regressions. Standard errors calculated allowing for clustering at the birth-year-community level (64 clusters) are shown in parentheses below coefficient estimates, which are in bold if significant at 5 % or lower

**Table 3:** Estimated impacts of pre-school, school, and post-school experiences on cognitive skills (*N* = 1,448)

Life-cycle stage	Representation	Set 1A		Set 1B		Set 2		Set 3		
		OLS	IV-GMM	OLS	IV-GMM	OLS	IV-GMM	OLS	IV-GMM	LIML
	Panel A: Verbal Z scores									
E_1_	HAZ score at age 6	**0.202** (0.028)	−0.071 (0.146)			**0.073** (0.021)	0.107 (0.103)	**0.073** (0.021)	0.184 (0.116)	0.089 (0.181)
E_2_	School attainment			**0.221** (0.006)	**0.101** (0.018)	**0.218** (0.006)	**0.105** (0.018)	**0.218** (0.006)	**0.087** (0.020)	**0.070** (0.028)
E_3_	Skilled job tenure							−0.001 (0.004)	**0.028** (0.014)	**0.029** (0.017)
	Age at interview in 2002–2004	**0.220** (0.094)	0.058 (0.119)	0.076 (0.075)	0.097 (0.054)	0.111 (0.074)	**0.173** (0.087)	0.111 (0.074)	**0.201** (0.095)	0.109 (0.126)
	Age at interview squared × 10	−**0.037** (0.014)	−0.014 (0.018)	−0.012 (0.012)	−**0.018** (0.009)	−0.018 (0.011)	−**0.029** (0.013)	−0.018 (0.011)	−**0.034** (0.014)	−0.020 (0.019)
	Constant	−2.690 (1.501)	−0.520 (1.710)	−2.164 (1.201)	−**1.709** (0.836)	−**2.574** (1.17)	−**2.781** (1.267)	−**2.585** (1.177)	−**2.993** (1.372)	−**1.582** (1.803)
	Test diagnostics									
	*F* Statistic	26.5	17.4	472.2	32.3	401.7	23.9	335.6	18.9	9.6
	[*p* value]	[< 0.01]	[< 0.01]	[< 0.01]	[< 0.01]	[< 0.01]	[< 0.01]	[< 0.01]	[< 0.01]	[< 0.01]
	Hansen *J* test [*p* value]		[0.03]		[0.17]		[0.12]		[0.16]	[0.19]
	Hausman test [*p* value]		[0.32]		[< 0.01]		[< 0.01]		[< 0.01]	
	Panel B: Nonverbal Z scores									
E_1_	HAZ score at age 6	**0.192** (0.034)	0.101 (0.128)			**0.108** (0.033)	0.035 (0.104)	**0.105** (0.034)	**0.329** (0.128)	0.333 (0.193)
E_2_	School attainment			**0.145** (0.007)	**0.125** (0.025)	**0.141** (0.007)	**0.127** (0.026)	**0.137** (0.007)	0.038 (0.036)	0.008 (0.048)
E_3_	Skilled job tenure							**0.018** (0.006)	**0.138** (0.018)	**0.149** (0.023)
	Age at interview in 2002–2004	**0.243** (0.090)	0.089 (0.104)	0.121 (0.102)	0.122 (0.085)	0.173 (0.097)	0.145 (0.086)	0.146 (0.097)	0.054 (0.099)	0.090 (0.119)
	Age at interview squared × 10	−**0.041** (0.013)	−0.019 (0.015)	−0.021 (0.015)	−0.021 (0.013)	−0.029 (0.015)	−0.025 (0.013)	−0.025 (0.015)	−0.015 (0.015)	−0.021 (0.017)
	Constant	−**3.032** (1.462)	−1.089 (1.597)	−**2.348** (1.674)	−2.260 (1.419)	−**2.956** (1.597)	−2.587 (1.397)	−2.513 (1.589)	−0.033 (1.590)	−0.451 (1.867)
	Test diagnostics									
	*F* Statistic	25.0	18.6	166.1	23.1	118.8	17.1	96.5	31.1	22.7
	[*p* value]	[<0.01]	[<0.01]	[<0.01]	[<0.01]	[<0.01]	[<0.01]	[<0.01]	[<0.01]	[<0.01]
	Hansen *J* test [*p* value]		[<0.01]		[<0.01]		[<0.01]		[0.12]	[0.12]
	Hausman test [*p* value]		[0.10]		[0.87]		[0.98]		[<0.01]	
	Panel C: First-stage diagnostics									
	Kleibergen-Paap Wald		7.28		15.41		6.35		6.31	
	F statistic									
	[*p* value]		[<0.05]		[<0.05]		[<0.05]		[<0.05]	

OLS, two-step instrumental variables GMM (IV-GMM), and limited information maximum likelihood (LIML) estimation using instruments in Table 2. Standard errors calculated allowing for clustering at the birth-year-community cohort level (64 clusters) are shown in parentheses below coefficient estimates, which are in bold if significant at 5 % or lower; *p* values are in brackets

**Table 4:** Estimated impacts of pre-school, school, and post-school experiences on verbal cognitive skills: alternative variable specifications (*N* = 1,448)

Life-cycle stage	Representation	Z scores Tobit	RCS + prelit IV-GMM	Quartiles IV-GMM
E_1_	HAZ score at age 6	0.097 (0.144)	0.870 (3.520)	**0.292** (0.133)
E_2_	School attainment	**0.099** (0.030)	**2.710** (0.569)	**0.086** (0.026)
E_3_	Skilled job tenure	0.025 (0.015)	0.699 (0.386)	**0.035** (0.014)
	Age at interview in 2000–2004	0.140 (0.109)	3.613 (3.024)	**0.208** (0.104)
	Age at interview squared 910	−0.025 (0.016)	0.658 (0.454)	−**0.034** (0.015)
	Constant	–	7.035 (43.28)	−0.436 (1.519)
	Test diagnostics			
	χ^2^ or *F* statistic	61.9	19.6	14.4
	[*p* value]	[<0.01]	[<0.01]	[<0.01]
	Hansen *J* test [*p* value]	–	[0.26]	[0.21]
	Hausman test [*p* value]	[<0.01]	[<0.01]	[<0.01]

Instrumental variables estimation using all instruments identified in Table 2. For Tobit, we report derivatives evaluated at the mean (d*P*/d*x*)), other results are IV-GMM as in Table 3. Standard errors calculated allowing for clustering at the birth-year-community cohort level (64 clusters) are shown in parentheses below coefficient estimates which are in bold if significant at 5 % or lower; p values are in brackets

**Table 5:** Estimated impacts of pre-school, school, and post-school experiences on cognitive skills: weighting for attrition (*N* = 1,448)

Panel A Life-cycle stage	Representation	Verbal skills IV-GMM	Nonverbal skills IV-GMM
E_1_	HAZ score at age 6	0.174 (0.122)	**0.296** (0.145)
E_2_	School attainment	**0.084** (0.023)	0.020 (0.041)
E_3_	Skilled job tenure	**0.037** (0.015)	**0.150** (0.021)
	Age at interview in 2002–2004	0.149 (0.098)	−0.016 (0.105)
	Age at interview squared 910	−0.025 (0.015)	−0.004 (0.015)
	Constant	−2.208 (1.415)	1.132 (1.702)
	Test diagnostics		
	*F* statistic	**16.1**	**27.8**
	[*p* value]	[<0.01]	[<0.01]
	Hansen *J* test [*p* value]	[0.16]	[0.13]

Panel B	First-stage diagnostics		
	
	Endogenous variable	*F* statistic on instruments excluded in second stage	[*p* value]

E_1_	HAZ score at age 6	**7.8**	[<0.01]
E_2_	School attainment	**12.9**	[<0.01]
E_3_	Skilled job tenure	**24.6**	[<0.01]
	Kleibergen–Paap Wald *F* statistic	**6.9**	[<0.05]

Instrumental variables (GMM) estimation, using all instruments identified in Table 2, and weighted as described in Sect. 4.2.4. Standard errors calculated allowing for clustering at the birth-year-community level are shown in parentheses below coefficient estimates, which are in bold if significant at 5 % or lower; *p* values are in brackets

## Footnotes

1 For example, there are hundreds of empirical studies that are interpreted as showing the effect of cognitive and other skills obtained through education on wages or incomes and the vast majority of them use school attainment to represent these skills (Psacharopoulos and Patrinos 2004). A smaller number of studies use direct measures of adult cognitive skills (including Boissiere et al. 1985; Murnane et al. 1995; Alderman et al. 1996; Glewwe 1996; LaFave and Thomas 2013). The many empirical studies of the effects of cognitive and other skills on outcomes such as health, nutrition, and fertility nearly all use school attainment to represent these skills (Strauss and Thomas 1998).

2 See references in the surveys by Strauss and Thomas (1995) and Behrman (2009).

3 See, for example Glewwe and Jacoby (1995), Engle et al. (2007), Heckman (2006a), Grantham-McGregor et al. (2007), and Victora et al. (2008).

4 There is considerable emphasis on post-school learning both “on-the-job” and through formal training programs. Standard earnings functions, whether motivated by a human capital investment models (e.g., Mincer 1974) or as hedonic price indices (e.g., Rosen 1974), generally include some measure of post-school work experience.

5 We are unaware of studies that estimate adult cognitive skills production functions, though there is work in which what might be called cognitive skills production functions for students are estimated as functions of inputs such as teacher training, student–teacher ratios, availability of books and other attributes of schools (see Hanushek 1996, as well as more recent work such as Todd and Wolpin 2003, 2007 and Cunha and Heckman 2008, Cunha et al. 2010). These studies do not consider prime-age adults, the endogeneity of inputs, or the impacts of pre-school or post-school experiences.

6 The vast majority of individuals in the sample started school by age seven (80 %) and completed formal schooling by age 15 (82 %).

7 An alternative production function specification is a value-added form in which the change in cognitive skills across stages (periods) is posited to depend on the level of cognitive skills at the end of the previous stage (Todd and Wolpin 2003, 2007). We do not have sufficient data to explore this specification.

8 That the pre-school experience encompasses all the effects of exogenous pre-school variables is a strong assumption that we relax in the empirical specifications. We make it here and in relation (8) to highlight the possible dependence of one life-cycle stage experience on a previous one.

9 If the correctly-specified regression model is *K*_3*v*_ = **Eβ_1_ + E_0v_β_2_** + *U*_3*v*_, where **E** is a vector with the three life-cycle stage experiences and **E_0v_** is a vector with unobserved endowments, then the standard omitted variable result is that *E*[**b_1_**] = **β_1_ + P_12_β_2_** where **P_12_** is the variance–covariance matrix between **E** and **E_0v_**. With certain structures of **P_12_**, this can lead to the first component of **b_1_** (for *E*_1_) being negatively biased and the second component of **b_1_** (for *E*_2_) being positively biased in OLS estimates.

10 This population also has been studied in the intervening years since the original survey, with particular emphasis on the impact of the nutritional intervention (Martorell et al. 2005) provide references to many of these other studies).

11 Most measures of attrition refer to households or individuals who were past infancy and early childhood when the sample was taken, so they do not include the effects of infant and early childhood mortality that account for over a quarter of the attrition in the data used for this study.

12 Respondents who reported having completed fewer than three grades of schooling, and those who reported three to five grades of schooling but could not read correctly the headline of a local newspaper article, were given a pre-literacy test that began with reading aloud single letters. They were considered literate if they passed the test with fewer than five errors out of 35 questions, the most difficult of which was reading aloud a five-word sentence.

13 The Raven’s scores that we use often are interpreted as if they represent innate abilities, perhaps genetically determined, and thus often are referred to as measures of “ability.” The term “skills,” in contrast, tends to be used to refer to capabilities that can be, or have been, affected by various experiences, such as education. In this paper, we directly explore factors that may influence the Raven; consequently, we refer to them as nonverbal skills to reflect their possible malleability as opposed to their measuring innate abilities.

14 If we were to approximate the function in relation (1v, 1n) with one indicator each for the three life-cycle stages in a second-order Taylor series expansion to allow diminishing marginal returns and interactions, we would need to estimate 9 parameters on endogenous variables. This more flexible specification exceeds the limits of what we are able to estimate with any precision.

15 Appendix A provides details of the construction of this variable.

16 We define skilled jobs to include white collar and administrative jobs, those with specialized skills (e.g., carpenters and mechanics), social service occupations (e.g., teachers, nurses) and own farm/own enterprise work that yields income in the top quintile for such activities in 2002–2004, under the assumption that such relatively large-scale enterprises required greater managerial and other skills to operate. Varying these definitions does not substantively change our results. Results are similar if we (1) treat as skilled labor only those with skilled wage employment; (2) use (1) along with a redefinition of skilled labor for agricultural work (based on planting a cash crop) and own business (based on the value of assets in the business); or (3) use the skilled years measure described in the text, but truncate it to 10 years to avoid the potential influence of outliers.

17 While we are unable to test whether total experience has a greater effect than recent experience, if there is depreciation of unused knowledge recent experience is likely to be more important than earlier experience.

18 Some of these shocks relate to the individuals’ parental families, particularly whether individuals’ parents were alive when individuals were at age 18. However, in contrast to when we include other parental characteristics (Sect. 4.3.2), these indicators can be different for different children within a household. Inclusion of the parental death shocks in the instrument set does not result in a rejection of the Hansen J overidentification test and leads to overall similar findings.

19 Standard corrections for clustering are valid only when the number of groups or clusters is large (Wooldridge 2003). Therefore, following Bertrand et al. (2004), we also estimated the models using block bootstrapped standard errors, using the same 64 clusters and resampling 10,000 times. Standard errors calculated from this approach were typically slightly larger than those reported in the paper, but significance patterns the same as our preferred IV-GMM specification in Table 3).

20 Using the 5 % critical value of 5.78 presented by Stock and Yogo (2005; Table 5), with a Kleibergen-Paap (KP) Wald *F* statistic (Kleibergen and Paap 2006), we reject the hypothesis that the instruments are weak, where weak in this case means having bias in the IV-GMM results that is larger than 20 % of the bias in the OLS results. To the extent that our estimates are biased, however, conditional on the validity of the excluded instruments, they are biased toward the OLS estimate, suggesting that the results we report are conservative and *understate* the differences between OLS and IV-GMM.

21 The Hansen *J* (HJ) statistic for overidentification does not reject the null hypothesis that the overidentifying restrictions are valid (i.e., that the model is well-specified and the instruments do not belong in the second-stage equation) at usual significance levels. Failure to reject the null hypothesis for the Hansen test is evidence that if any one of the instruments is valid, so are the others. Since the instrument set includes the randomly allocated exposure to the intervention and the earthquake indicator, both of which are likely to be valid, we interpret this as evidence in support of the validity of all the instruments. Further supporting this is the finding that in models in which we include additional parental characteristics (Sect. 4.2.2), the overidentification test fails, indicating that the HJ test has sufficient power to reject with these data.

22 Increases in the estimated effect of HAZ at age six and skilled job tenure after we instrument likely also reflects some random measurement error bias, although the approximate tripling of the estimated coefficient is not likely to be due solely to measurement error.

23 As for pre-school experience described above, the increase in this coefficient estimate after instrumenting is likely in part, but not entirely, due to random measurement error.

24 We also considered three-stage least squares estimates (with bootstrapped standard errors allowing for clustering at the birth cohort level) and they yield similar results.

25 We also considered estimating relations for women and men separately, but results are imprecise as we halve the sample size for each analysis.

26 For these estimates, we augment the instrumental variable set with two additional variables—an interaction between male and each of the exposure to the nutritional intervention variables, since there is evidence that the intervention affected males and females differently (Maluccio et al. 2009).

27 These include mother’s and father’s school attainment and a household wealth index. The wealth index is constructed using data on a set of household durables and housing characteristics observed in the early 1970s. Using principal components, these assets and characteristics were combined into an index that we call a “wealth” index (Maluccio et al. 2005).

28 For RCS + pre-literacy score, we sum the raw score from the pre-literacy screen and the reading and comprehension tests under the assumption that those who were exempt from the pre-literacy test would have earned a perfect score had they taken it. Since those who failed the pre-literacy test have scores in the 0–35 range, this leads to variation in scores among those to whom we assigned zero for the RCS alone. For the quartile measure, each individual’s reading and comprehension test score is recorded in the quartile of the distribution in which it falls. All those who failed the pre-literacy test (17 %) are in the first (lowest) quartile.

29 Stein et al. (2008) present a related finding that the nutritional intervention had significant impacts on adult cognitive skills even with controls for school attainment (although they do not attempt to control for the determinants of school attainment). Maluccio et al. (2009) also present a related finding that reduced-form estimates of adult cognitive skills indicate significant and substantial effects of the nutritional intervention.

## Appendix A Data appendix for pre-school HAZ score indicator

The data include from one to 15 measurements of height-for-age Z scores on each of 1,954 children from the original sample between 1969 and 1977 (WHO 2006). Not all of these individuals, however, were measured at the same ages or at any particular given age. For example, the greatest number of individuals was measured at nine months (8.5 m–9.5 m)—951 children or 49 % of those ever measured as infants and children. Because of the tendency for similar age patterns in the Z scores for a poorly nourished population such as this one (Martorell 1997), we use this information to obtain an estimate of the height-for-age Z score for individuals in the sample at a common pre-schooling age. For children in this sample, in the early months of life there is a tendency for a sharp drop in Z scores for height-for-age that then levels off and reaches a minimum at about 30 months of age, after which it increases slightly and approaches an asymptote just below −2.0 (the common cutoff for stunting) throughout the remainder of the pre-schooling period. Based on our objective of summarizing the entire pre-schooling experience, we use the height-for-age Z score at age 72 months (6 years) as our indicator of pre-schooling experience, which is both close to the age of starting school and an age where Z scores are relatively stable.^30^ Since this measure is not available for the entire sample, when it is missing we estimate it using measurements of the Z score for the child at ages other than 72 months. We first estimate the Z score-age relation with dummy variables for age categories^31^ other than 72 months, controlling for child-level fixed effects and then use the estimates of the age category dummy variables to adjust the nearest observed measurement of each child (for whom we do not have an observation at age 72 months) by the average difference between the measurement at the observed age and at 72 months.^32^

Even though the data permit the estimation of height-for-age Z scores at age 72 months for 1,954 individuals in the sample as compared with 1,448 for whom we have both adult cognitive skills test scores, for 180 individuals (12 %) for whom we have the test scores we do not have information with which to estimate the height-for-age Z score at age 72 months. For the 1,268 individuals for whom we have an actual or predicted height-for-age Z score at age 72 months, the mean value is −2.24 (median −2.22), almost at the cutoff for the definition of stunting, with a SD of 0.96. The means do not differ significantly for males versus females. To retain the 180 observations on individuals without pre-schooling height-for-age when estimating the impact of the experiences during the schooling and post-schooling periods, we replace the missing height-for-age Z score with the sample median of −2.22. Removing these observations leads to similar, and more highly significant results to those in the paper.

## Appendix B

See Table 6.

**Table 6:** Attrition probits to construct weights used in Table 5, reweighting for attrition bias (*N* = 2,392)

	Model 1	Model 2
Covariates	(1) if in sample	(1) if in sample
Male	−**0.109** (0.019)	−**0.121** (0.021)
Age at interview in 2002–2004	−**1.780** (0.168)	−**1.690** (0.162)
Age at interview in 2002–2004 squared	**0.020** (0.002)	**0.019** (0.002)
Twins	−**0.187** (0.081)	−**0.235** (0.082)
Student–teacher ratio at age 7	0.002 (0.003)	0.002 (0.002)
Lower secondary school available at age 7	0.044 (0.045)	0.047 (0.045)
Born in the two years prior to 1976 earthquake	0.097 (0.061)	0.043 (0.062)
Mother or father had died by age 18	0.091 (0.046)	**0.163** (0.042)
Ln Salary in manufacturing at age 18	−**4.031** (0.263)	−**3.817** (0.264)
Exposure to intervention 0–36 months	0.039 (0.069)	0.027 (0.071)
Exposure to intervention 0–36 months × *atole*	0.023 (0.061)	−0.013 (0.058)
Child lived with both mother and father in 1975	–	**0.077** (0.038)
Child lived with both mother and father in 1987	–	**0.058** (0.027)
Mother alive in 2002	–	**0.254** (0.042)
Father alive in 2002	–	**0.120** (0.031)
Mother living in original community in 2002	–	**0.094** (0.037)
Father living in original community in 2002	–	0.046 (0.031)
Number of siblings in survey	–	−0.002 (0.002)
Whether any sibling reinterviewed in 2002–2004	–	**0.269** (0.043)
χ^2^ statistic on variables in model 2 only	–	**312.6** [< 0.01]
Model χ^2^ statistic	**328.9** [< 0.01]	**618.5** [< 0.01]
Pseudo-*R*^2^	0.22	0.30

Sample consists of all 2,392 individuals who were exposed to the supplementation intervention between 1969 and 1977, 1,448 of whom are included in the main analyses. Standard errors calculated allowing for clustering at the birth-year-community level are shown in parentheses below coefficient estimates, which are in bold if significant at 5 % or lower; *p* values are in brackets (StataCorp 2011). Derivatives evaluated at the mean (d*P*/d*x*) presented
